# Cancer Cells Differentially Activate and Thrive on *De Novo* Lipid Synthesis Pathways in a Low-Lipid Environment

**DOI:** 10.1371/journal.pone.0106913

**Published:** 2014-09-12

**Authors:** Veerle W. Daniëls, Karine Smans, Ines Royaux, Melanie Chypre, Johannes V. Swinnen, Nousheen Zaidi

**Affiliations:** 1 KU Leuven – University of Leuven, Faculty of Medicine, Department of Oncology, Laboratory of Lipid Metabolism and Cancer, Leuven, Belgium; 2 Department of Oncology, Janssen Research and Development, a division of Janssen Pharmaceutica NV, Beerse, Belgium; 3 Microbiology and Molecular Genetics, University of the Punjab, Lahore, Pakistan; Clermont Université, France

## Abstract

Increased lipogenesis is a hallmark of a wide variety of cancers and is under intense investigation as potential antineoplastic target. Although brisk lipogenesis is observed in the presence of exogenous lipids, evidence is mounting that these lipids may adversely affect the efficacy of inhibitors of lipogenic pathways. Therefore, to fully exploit the therapeutic potential of lipid synthesis inhibitors, a better understanding of the interrelationship between *de novo* lipid synthesis and exogenous lipids and their respective role in cancer cell proliferation and therapeutic response to lipogenesis inhibitors is of critical importance. Here, we show that the proliferation of various cancer cell lines (PC3M, HepG2, HOP62 and T24) is attenuated when cultured in lipid-reduced conditions in a cell line-dependent manner, with PC3M being the least affected. Interestingly, all cell lines - lipogenic (PC3M, HepG2, HOP62) as well as non-lipogenic (T24) - raised their lipogenic activity in these conditions, albeit to a different degree. Cells that attained the highest lipogenic activity under these conditions were best able to cope with lipid reduction in term of proliferative capacity. Supplementation of the medium with very low density lipoproteins, free fatty acids and cholesterol reversed this activation, indicating that the mere lack of lipids is sufficient to activate *de novo* lipogenesis in cancer cells. Consequently, cancer cells grown in lipid-reduced conditions became more dependent on *de novo* lipid synthesis pathways and were more sensitive to inhibitors of lipogenic pathways, like Soraphen A and Simvastatin. Collectively, these data indicate that limitation of access to exogenous lipids, as may occur in intact tumors, activates *de novo* lipogenesis is cancer cells, helps them to thrive under these conditions and makes them more vulnerable to lipogenesis inhibitors. These observations have important implications for the design of new antineoplastic strategies targeting the cancer cell's lipid metabolism.

## Introduction

Rapidly proliferating cancer cells require a constant supply of lipids for membrane biogenesis and protein modifications. Several studies have shown that, in order to cope with these increased demands, cancer cells either increase their uptake of lipids or activate *de novo* lipid synthesis [1]–[5]. Enhanced fatty acid synthesis is found in 20% to 90% of tumors of many different types and is reflected in the up-regulation of key enzymes involved in this pathway [1]. These include fatty acid synthase (FASN), acetyl-CoA carboxylase alpha (ACACA) and ATP-citrate lyase (ACLY). Numerous reports indicate that the activation of these enzymes occurs downstream of growth factor signaling and other oncogenic events, irrespective of the presence of extracellular lipids [1], [6]–[12]. Also cholesterol synthesis, through the mevalonate pathway, is active in many cancer cells. Importantly, inhibition of fatty acid synthesis or cholesterol synthesis pathways by RNA interference or chemical inhibitors results in growth arrest of lipogenic tumor cells, both *in vitro* and *in vivo*, rendering these enzymes interesting targets for antineoplastic therapy [1], [13]–[19]. Unfortunately, the cytotoxic effects induced by inhibition of *de novo* lipid synthesis pathways appear to be averted by the presence of exogenous lipids or intermediate metabolites [13], [20], [21]. These observations suggest that it is the dependency on *de novo* lipid synthesis that determines the response of cancer cells to inhibition of these pathways and that extracellular lipids may compromise the therapeutic benefits of these inhibitors.

Here, to gain more insight into the complex interplay between exogenous lipids and *de novo* lipid synthesis pathways in cancer cells and to explore how this interplay may affect the efficacy of lipid-targeting antineoplastic therapies, we examined the impact of lipid deprivation on cell proliferation and the response to lipogenic inhibition in a variety of well-established lipogenic and less lipogenic cancer cell line models. Interestingly, we found that a lipid-reduced growth environment differentially affects the growth of cancer cell lines and is sufficient to turn on *de novo* lipogenesis pathways even in cancer cell lines that are considered non-lipogenic. This activation helps cancer cells to maintain their proliferation rate in a low-lipid environment and renders them more sensitive to lipogenesis inhibitors. These data re-emphasize the heterogeneity of cancer cells in terms of their metabolic requirements, they stress the importance of extracellular conditions and have important implications for the improved design of therapeutic strategies based on the manipulation of lipid requirements of tumor cells.

## Materials and Methods

### Cell culture and treatments

All cell lines were obtained from the American Type Culture Collection (ATCC). Cell culture reagents were purchased from Invitrogen unless stated otherwise. The PC3M cell line was cultured in HyClone MEM/EBSS medium (Thermo Scientific), supplemented with 10% fetal bovine serum (FBS), 100 mM Sodium Pyruvate, 10 mM Non-Essential Amino Acids, 2 mM L-Glutamine, 50 µg/ml Gentamicin and 1X BME Vitamins (Sigma). HOP62 cells were grown in RPMI 1640 supplemented with 10% FBS, 2 mM L-Glutamine and 50 µg/ml Gentamicin. HepG2 cells were cultured in MEM supplemented with 10% FBS, 2 mM L-Glutamine, 50 µg/ml Gentamicin, 100 mM Sodium Pyruvate, 0.37 g/L Sodium bicarbonate, 10 mM Non-Essential Amino Acids. The T24 cell line was cultured in DMEM medium, supplemented with 10% FBS, 2 mM L-Glutamine, 50 µg/ml Gentamicin. All the cell lines were grown in the atmosphere of 5% CO_2_ and 37°C. For lipid-reduced conditions the media were supplemented with 10% HyClone lipid-reduced FBS (Thermo scientific). Differences in lipids and related components in normal versus lipid-reduced FBS are listed in Supplementary Table S1. Simvastatin was purchased from Merck Sharp. Soraphen A was received from Dr. R. Jansen, Helmholtz-Zentrum f. Infektionsforschung, Mikrobielle Wirkstoffe, Braunschweig, Germany [22], [23]. Water-soluble cholesterol, glyceryl trilinoleate and glyceryl trilinolenate were purchased from Sigma. Very-low density lipoproteins (VLDL) were obtained from Merck Millipore. For culturing the cells in the presence of different fatty acids mixtures, palmitic (16∶0), oleic (18∶1), linoleic (18∶2), α-linolenic (18∶3), arachidonic (20∶4) and docosahexaenoic (22∶6) acid (Sigma) were complexed to fatty acid–free BSA (Sigma) as described previously [18], before addition to the culture medium. Triglycerides were incubated in normal or lipid-reduced FBS for 30 minutes at 37°C before addition to the culture media.

### Immunoblotting analysis

Equal amounts of proteins were loaded onto precast gels (NuPAGE, Invitrogen), transferred to PVDF membranes and incubated with antibodies against ACLY (monoclonal rabbit, ab40793, Abcam), FASN (monoclonal rabbit, 3180, Cell Signaling) and β-actin (monoclonal mouse, Sigma). The membranes were washed and probed with goat anti-rabbit conjugated with Alexa Fluor 680 (Invitrogen) secondary antibodies. Fluorescent signal was then measured using the Licor Odyssey system (LI-COR Biosciences).

### Proliferation assay

PC3M and HOP62 cells were seeded at a density of 5×10^4^ cells ml^−1^ in 24-well tissue culture plates (Nunc). T24 cells were seeded at 2×10^4^ cells ml^−1^ in a 24-well tissue culture plate. HepG2 cells were seeded at 2.5×10^4^ cells ml^−1^ in Poly-D-Lysine 96-well microplates, black/clear (BD Bioscience). The cells were seeded in normal or lipid-reduced medium. Growth curves were constructed by imaging plates using the *Incucyte system* (Essen Instruments), where the growth curves were built from confluence measurements acquired during round-the-clock kinetic imaging. For determining the cells number the cells floating in the culture medium were pooled together with the adherent cells after trypsinization. Cell were stained with trypan blue and counted using Countess automated cell counter (Invitrogen).

### RNA isolation and Real-time quantitative PCR (qPCR)

Total RNA from cultured cells was extracted and DNaseI treated using the RNeasy Mini kit (Qiagen) according to manufacturer's instructions. 3 µg of total RNA served as template for cDNA synthesis using Oligo dT primers and Superscript III reverse transcriptase in a volume of 20 µl during 1 hour at 50°C. This was followed by inactivation of the enzyme at 70°C for 15 minutes, according to the recommendations of the manufacturer (Invitrogen, Carlsbad, CA). Quantitative PCR (qPCR) was performed on an ABI Prism 7900-HT Sequence Detection System (Applied Biosystems) using a qPCR core kit w/o dUTP (Eurogentec). The thermal cycling conditions were 10 min at 95°C, followed by 45 cycles of 15 s at 95°C and 1 min at 60°C. Validated pre-designed Taqman Gene Expression Assays (Applied Biosystems) corresponding to the housekeeping genes TFRC (Hs00951083_m1) and PGK1 (Hs00943178_g1) were used to generate standard curves on serial dilutions of cDNA. The relative standard curve method was used to calculate the expression values. After normalization by TFRC or PGK1 the relative expression values were calculated. The other Taqman Gene Expression assays used in this study are: ACLY (Hs00982738_m1), ACSS2 (Hs00218766_m1), FASN (Hs01005622_m1), ACACA (Hs01046047_m1), HMGCR (Hs00168352_m1).

Total RNA from T24 cells was prepared using PureLink RNA Mini Kit (Ambion). The purity and concentration of RNA were assessed using a NanoDrop DM-1000 spectrophotometer (Nanodrop Technologies). 3 µg of RNA of each sample was used as template for cDNA synthesis using random hexamer primers and Superscript II reverse transcriptase, according to the manufacturer's protocol (Invitrogen). Primers were designed with Primer-BLAST software of NCBI, where possible primers spanning an exon-exon junction were selected. The specificity of the primers was checked by sequence analysis in the Primer-BLAST software and melt curve analysis. Quantitative PCR experiments were performed on a 7500 Fast Real-Time PCR system (Applied Biosystems) using the Fast SYBR Green Master Mix (Applied Biosystems). The thermal cycling conditions were 20 seconds at 95°C, followed by 40 cycles of 3 seconds at 95°C and 30 seconds at 60°C. The obtained Ct-values were normalized using 18S as a housekeeping gene.

### Apoptosis assay

Cells were seeded in normal or lipid-reduced growth conditions and were incubated and treated for 72 hours with Soraphen A or Simvastatin. Apoptosis was assessed by using the *Guava Nexin* kit and the *Guava PCA system* (Guava Techinnologies). The Guava Nexin assay utilizes two stains (annexin V and 7-amino actinomycin D [7-AAD]) to quantify the percentage of apoptotic cells. Cells that stain positive for both dyes are in the later stages of apoptosis, prior to cell death. The Nexin assay was performed according to the manufacturer's protocol. The data were collected and analyzed by a Guava personal cell analysis (PCA) flow cytometer with use of CytoSoft software (Guava Technologies). Approximately 2000 cells were analyzed.

### 3D cell culture

HepG2 cells were seeded in ultra-low attachment 96-well plates (Corning Costar) at a density of 750 cells/well/200 µl medium. Cells were seeded and maintained in normal or lipid-reduced growth medium and monitored for 20 days. After spheroid formation, 50% medium volume was replaced every 3–4 days. The spheroids were analyzed using *IN Cell Analyzer* (GE Healthcare).

### ATP assay

Viability of cells comprising the spheroids was determined by measurement of cell ATP content using CellTiter-Glo Luminescent Cell Viability Assay (Promega) according to manufacturer's protocol. Briefly, 100 µl ‘CellTiterGlo-reagent’ was added to the wells containing spheroids. Before adding the CellTiterGlo-reagent 100 µl medium was removed from each well. The spheroids are lysed by shaking for 10 minutes followed by pipetting. 100 µl of cell suspension from each well is transferred to a white Optiplate 96 (Perkin Elmer). Luminescence is measured.

### Lipid synthesis

Cells were cultured in 24-well plates in normal or lipid-reduced medium. After 72 hours [^14^C]-labeled acetate (56 mCi/mmol, 0.2 µCi/well, Amersham Biosciences) was added to the cells. After 4 hours of incubation the cells were collected by trypsinization and pelleted by centrifugation. Lipids were extracted using a modified Bligh Dyer method, as previously described [19].^14^C-incorporation into cellular lipids was quantitated by scintillation counting, using a Packard 1600CA *Tri-Carb liquid scintillation counter* (Packard Instrument Company). The obtained counts were normalized for sample DNA content, to take into account the differences in number of cells after 72 hours in lipid-reduced medium conditions.

### Nanofluidic proteomic analysis

The expression of the precursor and the active form of SREBP1 and SREBP2 was investigated by a size-based Simple Western immunoassay, using a Peggy Nanopro device (Protein Simple). The total protein concentration of the samples was determined using a BCA Protein Assay (Pierce). Equal amount of sample were prepared in Simple Western dilution buffer, reduced and denatured before loading onto the plate. Plates were prepared according to the manufacturing's procedure, using all reagents from Protein Simple. SREBP1 and SREBP2 antibodies (Active Motif, #39939 and #39941) were used at 1∶25 dilution, α-tubulin antibody (Cell Signaling, #2125) was used at a 1∶500 dilution. Data were analyzed using the Simple Western Compass software. Protein expression (area under the curve, AUC) was corrected for alpha-tubulin loading control (AUC SREBP/AUC alpha-tubulin).

### Statistical analysis

The results were analyzed by Students't-test (Excel) or one-way ANOVA followed by Tukey's multiple comparison test (GraphPad Prism Software), where applicable. P-values <0.05 were considered statistically significant. The data presented represent means ± S.D. as indicated in the corresponding figure legends.

## Results

### Lipid-reduced growth conditions differentially attenuate the proliferation rate of tumor cell lines

**Figure 1:**
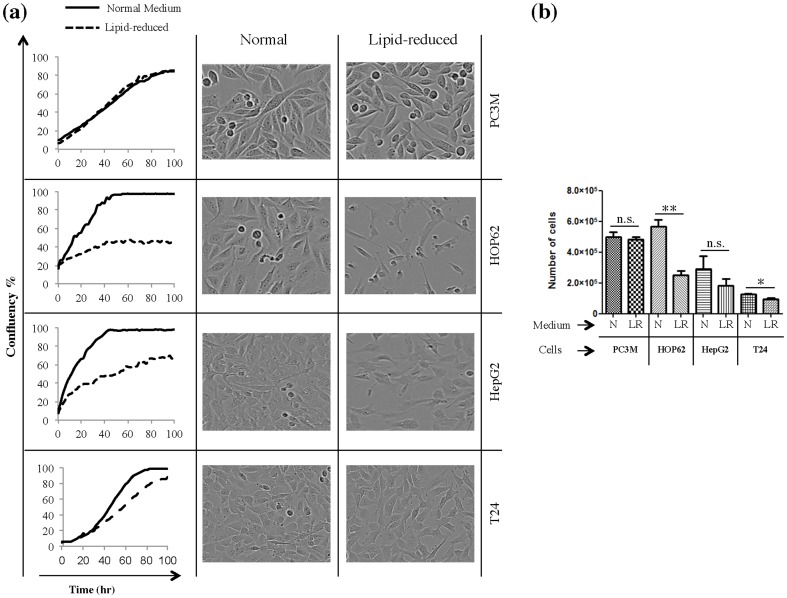
Lipid-reduced (LR) growth conditions attenuate 2D proliferation rate of HOP62, HepG2 and T24 cells but not of PC3M cells. **(a)** Proliferation curves for PC3M, HOP62, HepG2 and T24 cells. Cells were seeded and cultivated in normal or lipid-reduced medium and cell proliferation was monitored by *Incucyte real-time imaging.* The panels on the right side of each proliferation graph show the phase contrast image of the corresponding cell line in both conditions. Results are representative of three independent experiments. **(b)** The number of live cells was counted using a trypan blue dye exclusion method, after 72 hours of culturing in normal (N) or LR medium. *Significantly different (*p≤0,05; **p≤0,01; ***p≤0,001), n.s. not significant (p>0,05).

To study the effect of lipid reduction on proliferation and survival of cancer cells we selected PC3M, HOP62, HepG2 and T24 cell lines. The first three cell lines are reported to have a lipogenic phenotype in standard cell culture conditions with complete serum. We observed in our previous study that these cell lines were affected by environmental lipids [24]. This was unexpected, since lipogenic cell lines are thought to be mainly dependent on *de novo* lipogenesis for fulfilling their fatty acids requirement. The T24 cells display a low-lipogenic phenotype under these conditions and were incorporated as a control cell line [14], [24]–[26]. All these cell lines were cultivated under normal conditions in medium with 10% complete serum or with 10% lipid-reduced serum. Incucyte real-time imaging and trypan blue exclusion assays revealed that cultivation in lipid-reduced conditions attenuated cell proliferation of the various cell lines to a different degree. HOP62 showed a dramatic reduction in growth rates when cultivated under lipid-reduced growth conditions, followed by HepG2 **(** **Figure 1a-b** **)**. T24 cells were much less affected and PC3M cells were not influenced at all **(** **Figure 1a-b** **)**. However, the low-lipid environment did not induce apoptosis in the PC3M and HepG2 cell lines (**Supplementary Figure S1)**.

**Figure 2:**
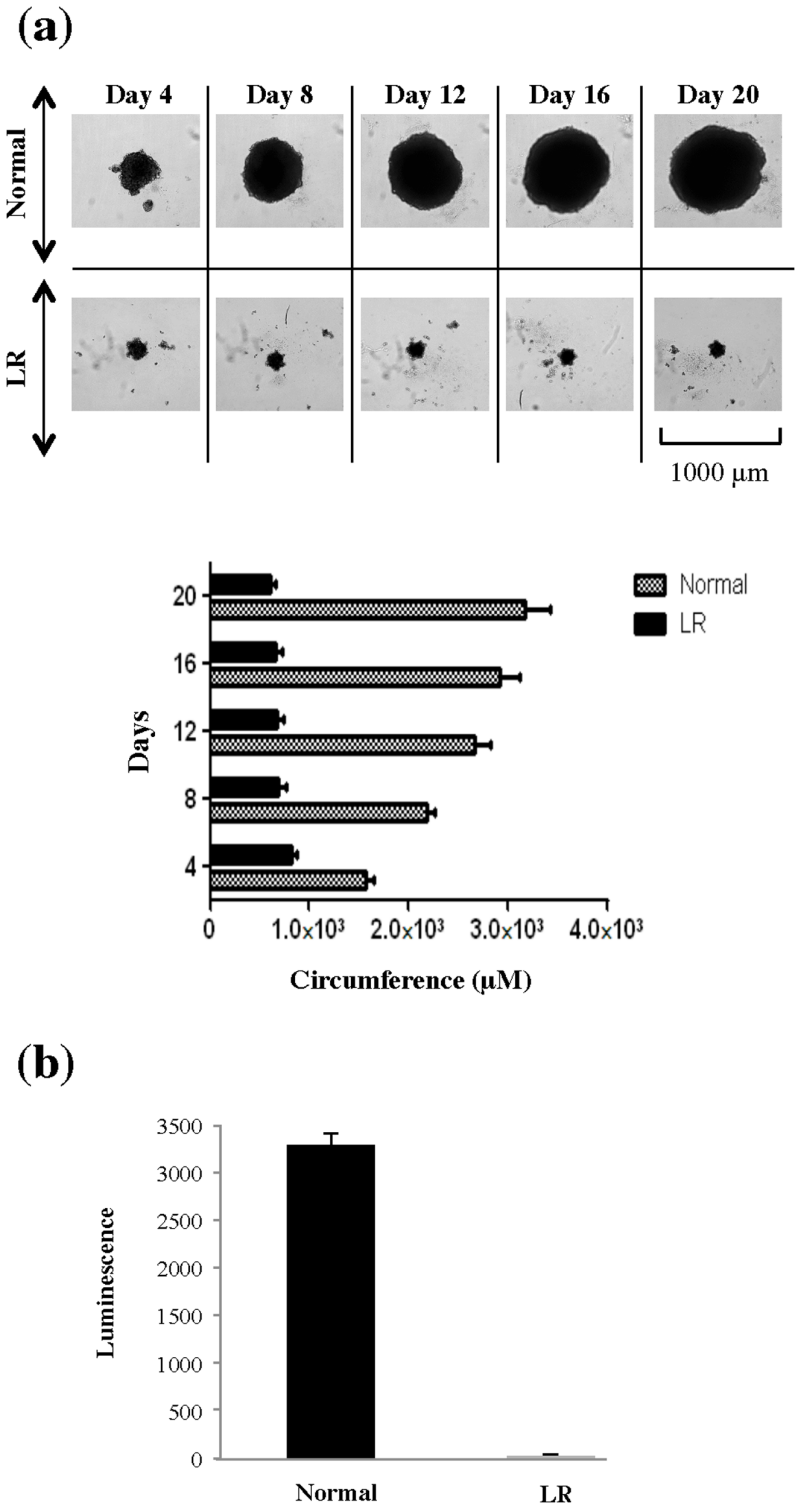
Lipid-reduced (LR) growth conditions stop 3D proliferation of HepG2. (**a**) Phase contrast microscopy showing HepG2-spheroid formation. HepG2 cells were seeded in normal and LR medium in ultra-low attachment plates at a density of 750 cells/well and cluster formation was monitored over twenty days as indicated. The spheroids were analyzed using an *IN Cell Analyzer 2000* instrument. Circumference of spheroids was measured using *IN Cell Analyzer 2000 software.* (**b**) Viability of cells comprising the spheroids was determined by measurement of cell ATP content using luminescence assay.

For HepG2, which have been previously shown to form compact 3D spheroids in 3D cell-culture systems [27]–[30], we also studied the effects of lipid-reduced conditions in a 3-dimensional (3D) cell culture system that mimics natural tissues and organs more closely than 2-dimensional (2D) cell culture system. In normal growth conditions a time-dependent increase in size of spheroids was observed **(** **Figure 2a** **)**. However, in lipid-reduced conditions HepG2-spheroid growth was completely arrested **(** **Figure 2a** **)**. At day 20, 5-fold larger spheroids were observed in normal growth conditions in comparison to lipid-reduced growth conditions **(** **Figure 2a** **)**. This was also reflected in the number of viable cells as revealed by ATP measurements [31] **(** **Figure 2b** **)**.

### Cancer cells turn on *de novo* lipid synthesis pathways in lipid-reduced growth conditions

**Figure 3:**
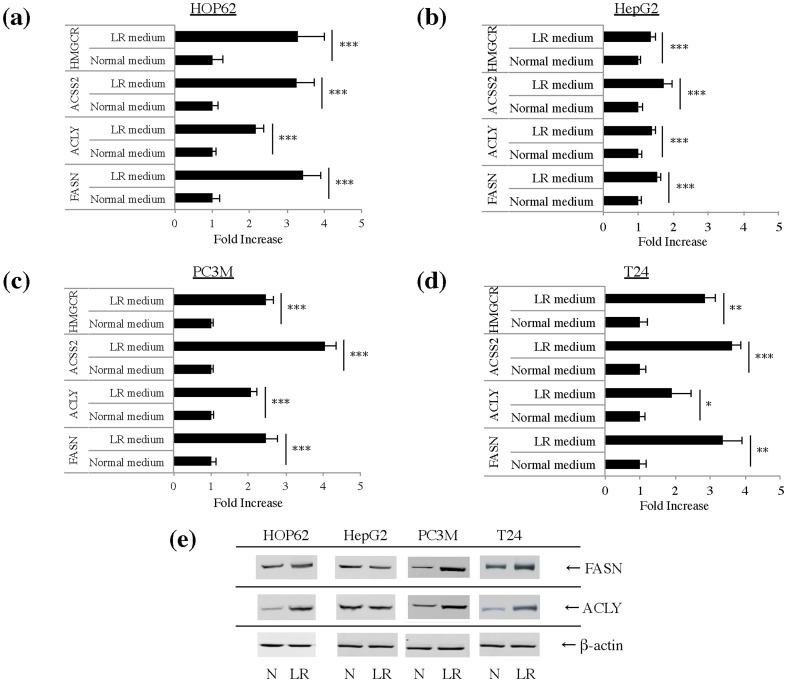
Lipid-reduced (LR) growth conditions differentially increase expression of FASN, HMGCR, ACLY and ACSS2 in different cancer cell lines. Gene expression of FASN, ACLY, ACSS2 and HMGCR was analyzed by qPCR analysis in **(a)** HOP62 **(b)** HepG2 **(c)** PC3M **(d)** T24 cells. Cells were cultivated in normal or LR medium for 48 hours. Data are expressed as mean ± S.D of triplicate samples, normalized to TFRC for HOP62, HepG2 and PC3M or to 18S for T24. *Significantly different (*p≤0,05; **p≤0,01; ***p≤0,001), n.s. not significant (p>0,05). **(e)** FASN and ACLY expression at protein level was analyzed by Western blot analysis in HOP62, HepG2, PC3M and T24 cells cultivated under normal (N) or LR medium for 72 hours. Beta-actin was used as a loading control.

**Figure 4:**
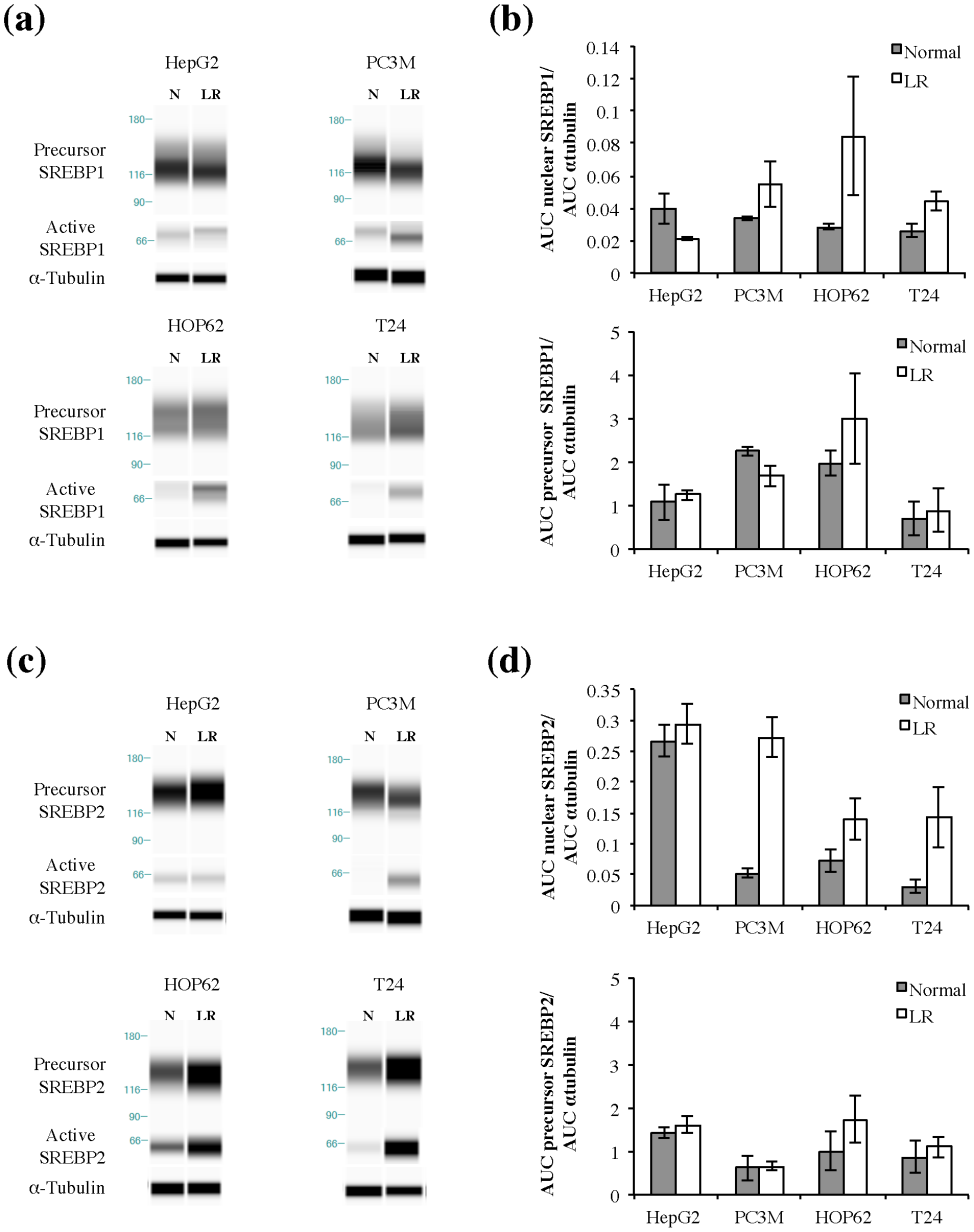
Lipid-reduced (LR) medium conditions increase the activation of SREBP1 and SREBP2 in some but not all cancer cell lines. **(a)** Representative virtual blot of Simple Western analysis of precursor and active SREBP1 expression in HepG2, PC3M, HOP62 and T24 cells after 72 hours cultivation in normal (N) or LR medium conditions. Alpha-tubulin is used as a loading control. Different exposures of precursor and active SREBP1 are shown in order to have an accurate exposure for both forms. For original data see Supplementary Figure S3a-d. **(b)** Quantitative analysis of Simple Western. data, expressed as area under the curve (AUC). Expression of SREBP1 was corrected for the loading control alpha-tubulin. Graph represents mean ± S.D. (n = 2–3). **(c)** Representative virtual blot of Simple Western analysis of precursor and active SREBP2 expression in HepG2, PC3M, HOP62 and T24 cells after 72 hours cultivation in normal (N) or LR medium conditions. Alpha-tubulin is used as a loading control. Different exposures of precursor and active SREBP2 are shown in order to have an accurate exposure for both forms. For original data see Supplementary Figure S3e-h. **(d)** Quantificative analysis of Simple Western. data, expressed as area under the curve (AUC). Expression of SREBP2 was corrected for the loading control alpha-tubulin. Graph represents mean ± S.D. (n = 2–3).

Cell proliferation in the lipid-reduced conditions is expected to depend on the ability of cancer cells to synthesize the required lipids *de novo*. However, our aforementioned proliferation data did not match the lipogenic activity of the cancer cell lines grown under standard conditions, as the growth rate of low-lipogenic T24 cells was less affected than the ones of the lipogenic HOP62 and HepG2 cells. Therefore, we assessed the ability of the cell lines to activate this pathway in lipid-reduced conditions. First, we checked the effect of normal and lipid-reduced growth conditions on mRNA expression profiles of major genes in *de novo* lipogenesis pathways: ATP-citrate lyase (ACLY), a cytosolic enzyme that catalyzes the generation of acetyl-CoA for both fatty acid and cholesterol synthesis, acyl-CoA synthetase short-chain family member 2 (ACSS2), which catalyses the synthesis of acetyl-CoA from acetate, fatty acid synthase (FASN), the key enzyme involved in fatty acid synthesis and hydroxymethyl glutaryl-CoA reductase (HMGCR), the rate-limiting enzyme in cholesterol synthesis. Ct-values of the tested genes are indicated in **Supplementary Table S2**. Interestingly, the expression of these enzymes was increased in all cell lines, including the non-lipogenic cell line T24, although to a somewhat different degree, with HepG2 being the least responsive (**Figure 3a-d**). Western blot analysis of FASN and ACLY showed that these enzymes were most dramatically up-regulated in lipid-reduced medium in PC3M and least in HepG2 (**Figure 3e**). In line with these findings, sterol regulatory binding protein 1 (SREBP1) and SREBP2, which are the main regulators of the expression of genes of the fatty acid synthesis and the mevalonate pathway respectively, also showed an increased expression **(Supplementary Figure S2)** and were further activated at the protein level in lipid-reduced conditions in HOP62, PC3M and T24 cells but not in HepG2 **(** **Figure 4** **a-d and Supplementary Figure S3)**. The carbohydrate-responsive element binding protein (ChREBP), another important regulator of the expression of lipogenic genes [32], was only expressed in detectable levels in the HepG2 cells (liver cancer cell line) (**Supplementary Figure S4a**) and in this cell line, we could not observe an activation and nuclear translocation of the transcription factor, when cultured in low-lipid conditions (**Supplementary Figure S4b**). In the PC3M, the HOP62 and the T24 cell lines, prostate, lung and kidney cancer cell lines respectively, we could not detect ChREBP in total cellular extracts or in the nuclear fractions, when cells were cultured in lipid-reduced growth conditions (**Supplementary Figure S4b**). These observations suggest that ChREBP does not play a pivotal role in the increased lipogenic gene expression in lipid-reduced conditions.

**Figure 5:**
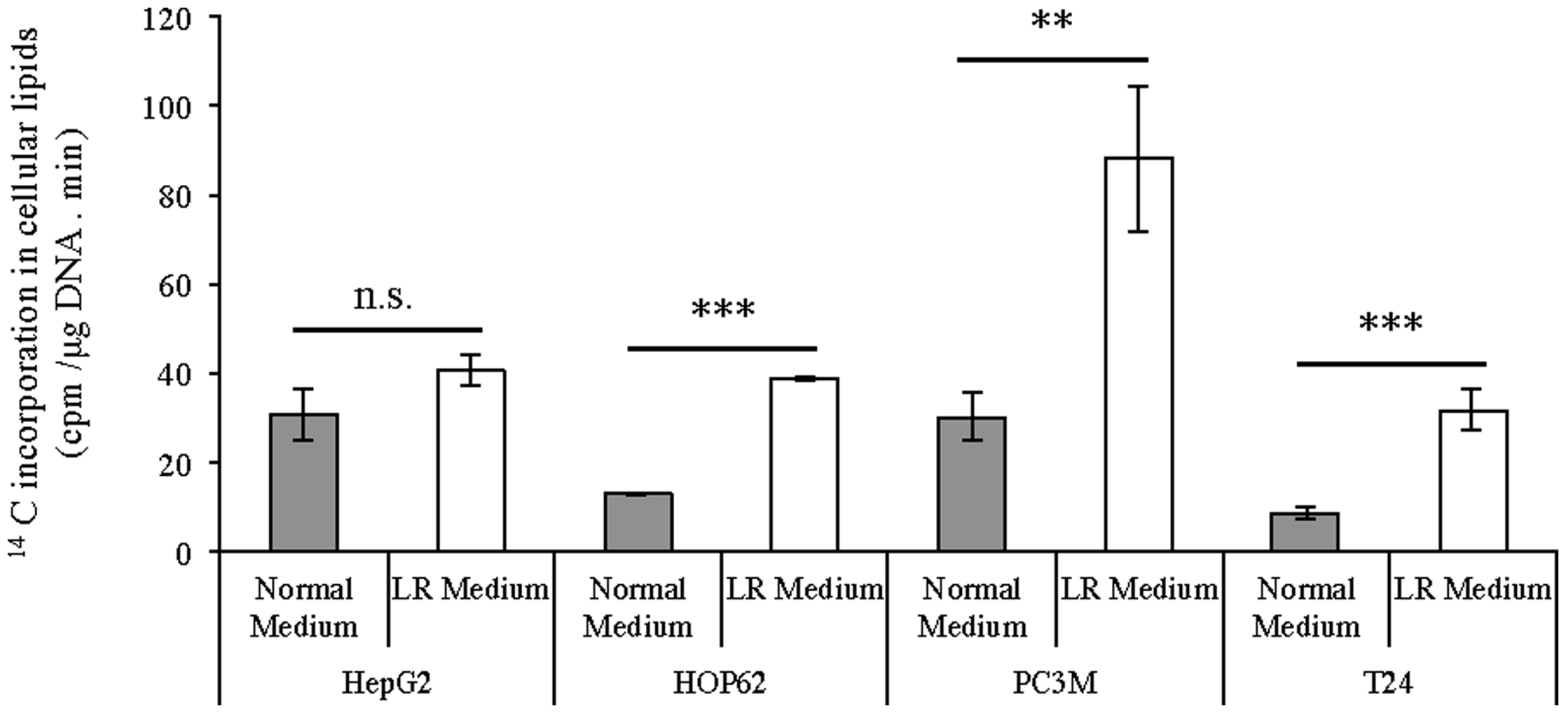
Lipid-reduced (LR) growth conditions affect lipid biogenesis in cancer cells. HepG2, PC3M, HOP62 and T24 cells growing in normal or LR medium for 72 hours, were incubated for the last 4 hours with ^14^C-acetate. Cellular lipids were extracted and the incorporation of ^14^C in the cellular lipids was determined by scintillation counting. Scintillation counts were normalized for sample DNA content. Results are representative of three independent experiments. *Significantly different (*p≤0,05; **p≤0,01; ***p≤0,001), n.s. not significant (p>0,05).

To corroborate our findings on increased expression of lipogenic genes in lipid-reduced conditions, lipid synthesis was determined by measuring the incorporation of radiolabeled acetate into cellular lipids. In line with our other observations, ^14^C-acetetate incorporation was significantly increased in the PC3M, HOP62 and T24 cells, when they were cultivated in a low-lipid environment (**Figure 5**). HepG2 cells, which already show a high basal lipogenic activity, did not display significant up-regulation of their lipid synthesis when grown in lipid-reduced growth medium. Although, HOP62 and T24 could up-regulate their *de novo* lipogenesis, they could only reach the same level of lipogenic activity as the HepG2 cells. By contrast, PC3M cells could up-regulate their lipogenic activity to a much higher level than the other cells lines. This gives an indication why these later cells could keep a normal growth rate in lipid-reduced conditions and the other three cell lines could not. These findings indicate that in lipid-deprived conditions cancer cells, irrespective of whether they are lipogenic or non-lipogenic in standard culture conditions, have the ability to up-regulate *de novo* lipogenesis pathways, be it to a different extent. The extent to which the cells up-regulate their lipogenic pathway in a lipid-reduced environment determines their ability to keep proliferating under these conditions.

### Up-regulation of *de novo* lipid synthesis induced by low-lipid environment can be reversed by VLDL and can be attributed to a shortage of fatty acids and cholesterol

**Figure 6:**
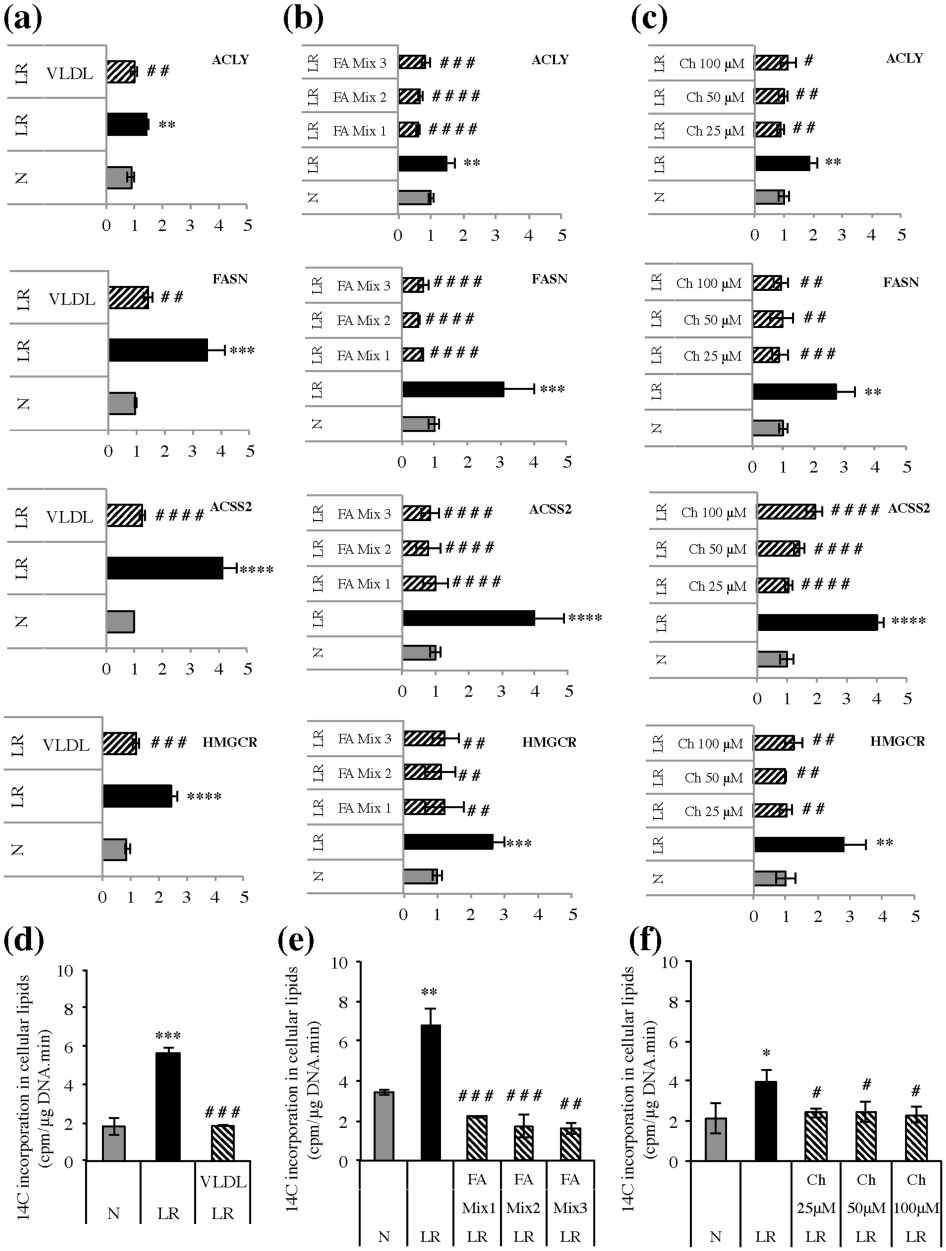
Addition of very-low density lipoproteins (VLDL), fatty acids and cholesterol to lipid-reduced (LR) growth conditions reverses the increased activation of the lipogenic pathway. Gene expression of FASN, ACLY, HMGCR and ACSS2 was analyzed by qPCR in T24 cells were cultured for 48 hours in normal (N) or LR growth conditions in the presence or absence of VLDL **(a)**, different fatty acid mixtures **(b)** and different concentrations cholesterol **(c)**. VLDL was added at a concentration of 607 µg triglycerides/ml serum (corresponding to the concentration triglycerides in normal FBS). Fatty acid (FA) mixtures were as follows, FA Mix 1: 20 µM linoleic (18∶2), 20 µM α-linolenic (18∶3), 5 µM arachidonic (20∶4), 5 µM docosahexaenoic acid (22∶6), FA Mix 2: 10 µM 18∶2, 15 µM 18∶3, 10 µM 20∶4, 15 µM 22∶6 and FA Mix 3: 20 µM 18∶2, 20 µM 18∶3, 5 µM 20∶4, 5 µM 22∶6, 30 µM oleic acid, 30 µM palmitic acid. Different cholesterol (Ch) concentrations are as indicated in the figures (25 µM, 50 µM or 100 µM). Data are normalized to 18S and represented as mean ± S.D. (triplicate per experiment and n = 3). Significance was determined by one-way ANOVA followed by Tukey's multiple comparison test. *Significantly different (*p≤0,05; **p≤0,01; ***p≤0,001; ****p≤0,0001) from normal medium control. ^#^Significantly different (^#^p≤0,05; ^##^p≤0,01; ^###^p≤0,001;^ ####^p≤0,0001 ) from LR control. **(d, e, f)** ^14^C-incorporation into cellular lipids was determined in T24 cells, cultured for 48 hours in normal (N) or LR growth conditions in the presence or absence of VLDL **(d)**, different fatty acid mixtures **(e)** and different concentrations cholesterol **(f)** as mentioned in (a, b and c). During the last 4 hours ^14^C-acetate was added and the incorporation of radioactivity in cellular lipids was normalized to sample DNA content. Representative experiment is shown, experiment was repeated two times. Significance was determined by one-way ANOVA followed by Tukey's multiple comparison test. *Significantly different (*p≤0,05; **p≤0,01; ***p≤0,001; ****p≤0,0001) from normal medium control. ^#^Significantly different (^#^p≤0,05; ^##^p≤0,01; ^###^p≤0,001; ^####^p≤0,0001) from LR control.

Next, we sought to confirm that the induction of *de novo* lipogenesis under lipid-reduced conditions was due to specific depletion of lipids from the growth medium. As lipid-reduced serum is prepared by removal of lipoproteins such as very-low density lipoproteins (VLDL) [33], which are a rich source of triglycerides and cholesterol, explaining the strong reduction of these two lipids in our lipid-reduced FBS (**Supplementary Table S1**), T24 cells were cultured in lipid-reduced conditions and the medium was supplemented with VLDL. Addition of VLDL decreased the expression of SREBP1a, SREBP1c and SREBP2 (**Supplementary Figure S5a)**. Consequently, the expression of FASN, ACACA, ACLY, ACSS2 and HMGCR was also significantly decreased (**Figure 6a**). This decrease in expression of the lipogenic genes was accompanied by a decreased activity of the lipogenic pathway, as determined by ^14^C-acetate incorporation assay (**Figure 6d**). To define whether the triglycerides or the cholesterol present in the VLDL particle caused this lipogenesis-inhibitory effect, we supplemented the cells with triglycerides or cholesterol alone. Addition of triglycerides could not reverse the elevated lipogenesis observed in the T24 cultured in low-lipid growth conditions (**Supplementary Figure S6**). Free fatty acids, however, did result in a significant rescue. Different mixtures of saturated, mono- and poly-unsaturated fatty acids decreased the expression of SREBP1a, SREBP1c and SREBP2 (**Supplementary Figure S5b**). Consequently, the expression of FASN, ACACA, ACLY, ACSS2 and HMGCR was also significantly decreased after fatty acid addition (**Figure 6b**) and was accompanied by a decreased activity of the lipogenic pathway, as determined by ^14^C-acetate incorporation assay (**Figure 6e**). These results clearly indicate that exogenous fatty acids may affect the regulation of *de novo* lipogenesis also in cancer cells. In contrast with fatty acid addition, supplementation of the lipid-reduced growth medium with cholesterol did not lead to decreased mRNA levels of SREBP1a, SREBP1c or SREBP2 (**Supplementary Figure S5c**), yet still affected the mRNA levels of the downstream lipogenic genes, FASN, ACLY, ACSS2 and HMGCR (**Figure 6c**). This is in accordance with previous reports showing cholesterol to regulate SREBP activity at the post-translational level, rather than at the translational level [34]. The cholesterol-induced decrease in the expression of lipogenic enzymes was accompanied by a decreased activity of the lipogenic pathway (**Figure 6f**), indicating that also exogenous cholesterol influences the activity of the lipogenesis pathway in cancer cells.

### Lipid-reduced growth conditions increase the sensitivity of cancer cells to inhibitors of lipid synthesis pathways

**Figure 7:**
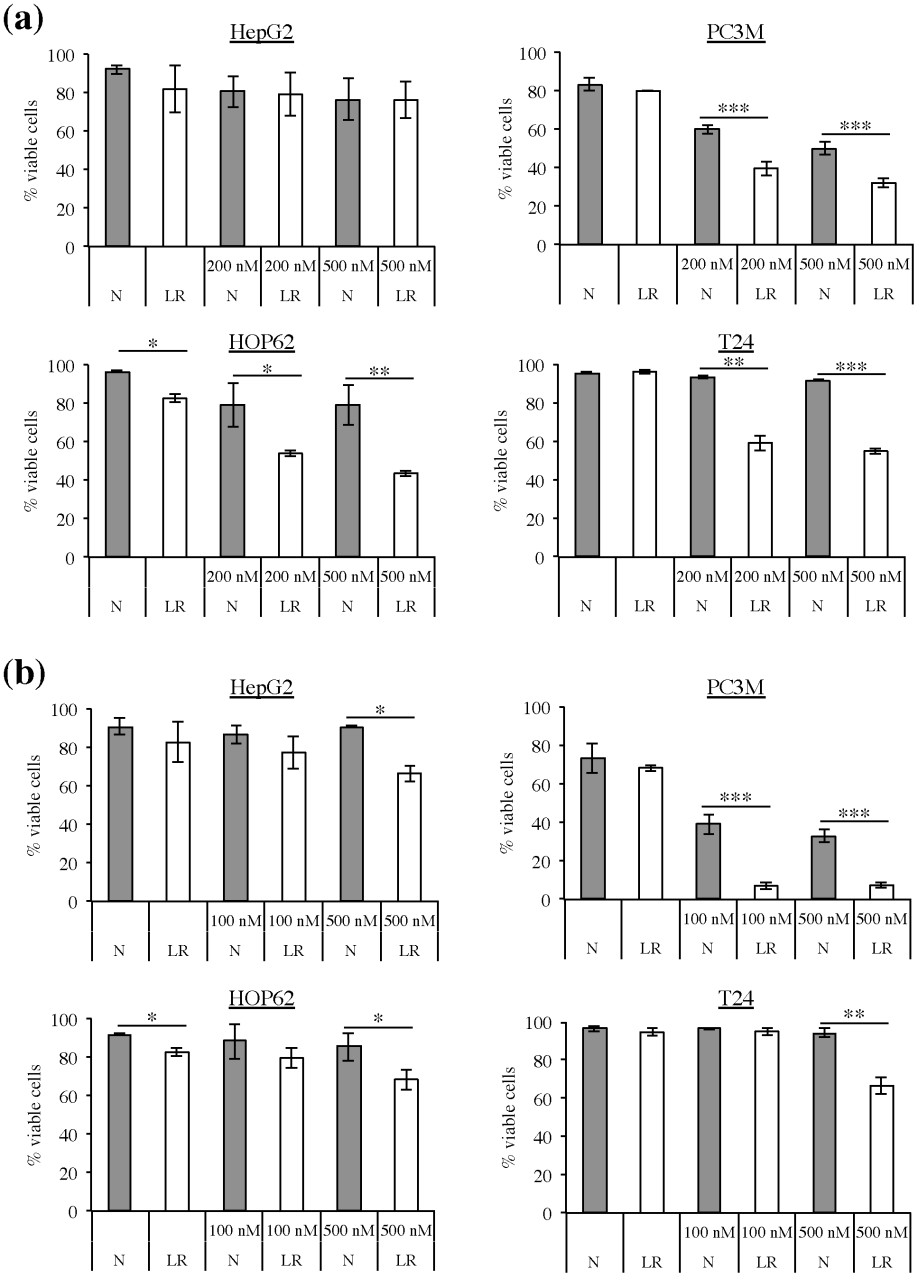
Lipid-reduced (LR) growth conditions increase the sensitivity of cancer cells to inhibitors of lipid synthesis pathways. HepG2, PC3M, HOP62 and T24 cells growing under normal (N) or LR growth conditions were treated with **(a)** Soraphen A (200 and 500 nM) and **(b)** Simvastatin (100 or 500 nM). Annexin V and 7-AAD staining was used to detect apoptotic cells after 72 hours of treatment (n = 3). Graph represents mean ± S.D. (n = 2–3). *Significantly different (*p≤0,05; **p≤0,01; ***p≤0,001) from normal growth condition.

To further evaluate the increased dependence of cancer cells on *de novo* lipid synthesis pathways in a low-lipid environment we determined the sensitivity of cancer cells to inhibitors of these pathways. Soraphen A is a highly potent inhibitor of ACACA, hence it blocks *de novo* lipid synthesis and induces apoptosis in cancer cells [13]. We observed that the sensitivity of PC3M, HOP62, and T24 cells to Soraphen A was markedly enhanced upon cultivation in lipid-reduced growth conditions. In these cell lines there was a significant decrease in the number of viable cells upon Soraphen A treatment in lipid-reduced growth conditions in comparison to normal growth conditions, but the extent of sensitization differed form cell line to cell line **(** **Figure 7a** **)**. The cell line that showed the highest increase in lipogenic activity when cultured under lipid-reduced conditions (**Figure 5**), PC3M cells, were the most sensitive to Soraphen A treatment in lipid-reduced conditions. HOP62 and T24 cells, two cell lines that increased their lipogenic activity to the same extent (**Figure 5**), also showed the same response to Soraphen A treatment in lipid-deprived conditions. Their response to Soraphen A was less pronounced than in PC3M cells, which is in accordance with their lower increase in lipogenic activity in lipid-reduced growth conditions. And finally, HepG2 cells, which don't increase their lipogenic activity in lipid-reduced growth conditions, did not show a better response to Soraphen A treatment when cultured in lipid-reduced conditions, compared to their response in normal growth conditions.

We also assessed the effect of simvastatin, an inhibitor of the mevalonate/cholesterol synthesis pathway on cancer cells in lipid-reduced and normal growth conditions. Low-lipid conditions also increased the sensitivity of cancer cells to this compound. There was a significant decrease in the number of viable cells upon treatment with simvastatin in lipid-reduced growth conditions in comparison to normal growth conditions **(** **Figure 7b** **)**. Again, a differential response to the treatment in lipid-reduced conditions was observed, with PC3M responding better than HOP62 and T24 and with the HepG2 cells responding the least. This differential response was in accordance with the degree of increased lipogenic activity of these cells in lipid-reduced growth conditions.

## Discussion

It is commonly accepted that cancer cells, in contrast to their normal counterparts, obtain the bulk of the required lipids from *de novo* lipogenesis, irrespective of the presence of exogenous lipids [1], [6], [7]. These observations channeled intense research towards the development of novel anti-cancer strategies based on inhibition of *de novo* lipogenic pathways. Recently, however, evidence is mounting that exogenous fatty acids may have more adverse effects on these strategies than originally thought. In cell cultures, exogenous fatty acids can functionally substitute for endogenously derived fatty acids in promoting cell viability and attenuate the cancer-specific toxic effect of lipogenesis inhibitors [13], [20], [21]. It has been recently reported that certain cancer cells such as aggressive triple-negative breast cancer cell lines express markers of lipolysis (lipoprotein lipase, LPL) and exogenous fatty acid uptake (CD36), concomitantly with markers of *de novo* lipogenesis (FASN) [4]. Along the same lines, clinical breast tumor specimens appear to universally co-express LPL and FASN irrespective of their biomarker status [4]. LPL expression in other tumors, such as liposarcoma and prostate cancer, also indicates that the metabolic machineries for both lipogenesis and lipolysis are widely co-expressed in cancers of diverse origins. These observations are compatible with an expanded metabolic repertoire for both the lipolytic and lipogenic generation of fatty acids in these tumors from exogenous and endogenous precursors, respectively. A recent work by Kuchiba *et al.* suggests that obesity is associated with an increased risk of FASN-negative colorectal cancers [35]. In contrast, no statistically significant association between obesity and risk of FASN-positive colorectal cancers was observed. Hence, in obese individuals cancer cells are less likely to up-regulate the lipogenic pathway and may rather acquire fatty acids from the circulation. These observations suggest that cancer cell's decision to adapt lipolysis or lipogenesis as a principal source of fatty acids may depend upon the metabolic status of the host. Moreover, the efficacy of targeting the lipogenic pathway as an antineoplastic therapy will depend on the reliance of individual tumors on *de novo* lipid synthesis. Taken together these observations underscore the importance of studying the effect of exogenous lipid availability on endogenous lipid synthesis pathways in cancer cells.

In the present study, to better understand the interrelationship between *de novo* lipid synthesis and exogenous lipids and their respective role in cancer cell proliferation and therapeutic response to lipogenesis inhibitors, we have cultured cancer cells in lipid-reduced conditions. Interestingly, we observed that the proliferation rate in different cancer cell lines was affected to varying extents. One of the tested cell lines i.e. PC3M was not affected at all by these conditions in terms of proliferation rate. In contrast, HOP62 and HepG2, which are also considered to be lipogenic similar to PC3M, showed a dramatic decrease in their proliferation rates when cultured in lipid-reduced conditions. Surprisingly, the proliferation rate of the T24 cell line, a cell line with a low lipogenic phenotype in standard conditions, was much less affected by lipid-reduction. These findings suggest that it is not the lipogenic phenotype *per se* (at least in standard conditions) that determines growth in lipid-reduced conditions. To explain this conundrum, we measured the lipogenic activity of the various cell lines in lipid-reduced conditions. In contrast to the traditional paradigm, that the lipogenic phenotype of cancer cells is independent of the availability of exogenous lipids [1], [7], we observed that cancer cells cultivated under lipid-reduced conditions substantially up-regulated the expression of various genes involved in *de novo* lipid synthesis through a mechanism that involves activation of SREBPs, similar to what has been reported in non-malignant cells [34]. This activation led to an actual increase in the rate of *de novo* lipid synthesis, in all cell lines tested, albeit to a different level. This effect was only marginal in HepG2 cells, whose proliferation rate, as indicated above, was dramatically reduced in low-lipid conditions. These findings suggest that even though the HepG2 cells display a high basal lipogenic activity, this level was not sufficient to sustain their growth rate in lipid-reduced conditions. The HepG2 cells are known to secrete a major part of their *de novo* synthesized lipids. So even though they have a basal high lipogenic activity, this will not provide them with sufficient fatty acids for membrane synthesis. The HOP62, by contrast, could significantly up-regulate their *de novo* lipogenesis, although this increased lipogenic activity was not sufficient to maintain their normal proliferation rate in lipid-reduced conditions. Interestingly, T24 cells, that are considered non-lipogenic in standard conditions, also markedly up-regulated their *de novo* lipogenic activity to a level that could partially support their growth in lipid-deprived conditions. PC3M cells, that were able to maintain their proliferation rate in lipid-reduced conditions, had the highest lipogenic activity in these conditions. This suggests that these cells were able to cope with lipid-reduced growth conditions by sufficiently elevating their rate of *de novo* lipid synthesis. These findings additionally indicate that cancer cells, irrespective of their lipogenic rate in standard conditions are able to up-regulate their lipogenic activity. To demonstrate that the elevation of lipogenic activity in lipid-deprived conditions is mediated by lipids, we supplemented the lipid-reduced medium with very-low density lipoproteins, fatty acids and cholesterol and observed that this supplementation reversed the increase of expression and activity of lipogenic enzymes induced by the lipid-reduced conditions. Hence, our data unequivocally show that *de novo* lipid synthesis in cancer cells is affected by exogenous lipids. Moreover, we observed that lipid-reduction is sufficient to up-regulate lipogenesis even in non-lipogenic cancer cells. The absolute levels of lipogenic activity under these conditions is likely to result from both up-regulation by the lack of lipids as well as other previously reported oncogene-induced mechanisms of up-regulation of lipogenic enzymes, and may determine the ultimate ability of cancer cells to thrive under lipid-reduced conditions. These findings could have significant implications for the growth of tumor masses in real tumors within the human body and for their response to anti-lipogenic therapies. In fact, in rapidly growing tumors, tumor cells may have limited excess to exogenous lipids in comparison to the cells growing in cultures with plenty of exogenous lipids. Consistent with our previous report that cancer cells cultivated under lipid-reduced growth conditions are more sensitive to inhibition of *de novo* lipid synthesis pathways [24], we observed that lipid-reduced conditions render the cancer cells more sensitive to the inhibitors of either fatty acid synthesis or cholesterol synthesis. The sensitivity of cancer cells to lipogenesis inhibitors in lipid-reduced conditions was proportional to their increase in lipogenic activity when cultured in a low-lipid environment, with the PC3M cell line, which showed the highest increase in lipogenic activity in lipid-reduced conditions, giving the best response to Soraphen A and simvastatin treatment under these conditions. The HepG2 cell line, which did not increase their lipogenesis, was the least sensitized for anti-lipogenic treatment in lipid-reduced conditions. Hence, the limited access to environmental lipids in a natural environment may render tumor cells more sensitive to lipogenesis inhibitors than estimated in standard culture conditions.

In conclusion, the present study demonstrated that a lipid-reduced growth environment differentially attenuates proliferation of various cancer cell lines. These effects appear to depend on the ability of cancer cells to cope with the decrease in extracellular lipid content by further elevating their *de novo* fatty acid synthesis. Cancer cells that are able to sufficiently enhance their *de novo* lipid synthesis in lipid-reduced environment such as PC3M maintain their proliferation rate in these conditions. Moreover, we showed that culturing cancer cells in lipid-reduced conditions increases their sensitivity to lipogenesis inhibitors and that this increased sensitivity was proportional to the increase in lipogenic activity in these conditions. These findings demonstrate the relative importance of lipid uptake and endogenous lipid synthesis for cancer cell growth and survival. This knowledge could be helpful in designing new anti-tumor strategies based on manipulation of lipid requirements of tumor cells. The effectiveness of lowering the fat intake on the outcome of breast cancer patients treated with classical chemotherapeutics has already been shown in a randomized, prospective clinical trial conducted by Chlebowski and colleagues [36]. A more recent study by Kuemmerle *et al.* has shown the importance of lipolysis and circulating low density lipoproteins in the growth and survival of cancer cells after lipogenic inhibition [4]. They suggested the use of inhibitors of the lipolytic pathway that at the same time inhibit the *de novo* fatty acid synthesis, like Orlistat [37] and conjugated linoleic acid [38], [39], as new antineoplastic therapeutics. In the light of our and these previously obtained observations one can speculate that the administration of the lipogenic and lipolytic inhibitors in combination with drugs or diets that lower triglyceride/lipoprotein levels in the blood could serve as a promising therapeutic tool against cancer. Further *in vivo* studies aimed at elucidating the effect of these inhibitors in combination with dietary fat-restriction are required to clarify the therapeutic potential of anti-cancer strategies based on the manipulation of lipid requirements of tumor cells.

## Supporting Information

Figure S1 **Culturing cells in lipid-reduced (LR) growth conditions does not induce apoptosis.** PC3M and HepG2 cells were cultured for 72 hours in normal or LR growth conditions. Cell death was analyzed by flow cytometry as described in materials and methods (n = 3).
(TIF)

Figure S2 **Lipid-reduced (LR) growth conditions increase expression of SREBP1 and SREBP2 in T24 cells.** T24 cells were cultured for 48 hours in normal or LR growth conditions. Gene expression levels of SREBP1a, SREBP1c and SREBP2 were analyzed by qPCR analysis. Data normalized to 18S rRNA and represented as mean ± S.D. (triplicate per experiment and n = 3). *Significantly different (*p≤0,05; **p≤0,01; ***p≤0,001).
(TIF)

Figure S3 **Original data Simple Western analysis of SREBP1 and SREBP2 expression shown in** **Figure 4a and 4c** **.** Panels shown in Figure 4a and 4c are depicted here in panel **1**. They are composed of the original Simple Western bands shown in panel **2**. Above the original Simple Western panels is indicated which bands of the blot are used to compose Figure 4a and 4c. The active and precursor SREBP bands shown in Figure 4a and 4c were taken from the same samples, but with a different exposure. This was done in order to have and accurate detection of both the active and the precursor form. Panels **(a-d)** show composition of panel a of Figure 4 (SREBP1 data), panels **(e-h)** show composition of panel c of figure 4 (SREBP2 data).
(TIF)

Figure S4 **Lipid-reduced (LR) growth conditions do not change the expression and nuclear translocation of ChREBP.** (**a**) ChREBP expression at protein level was analyzed by western blot analysis in HepG2, PC3M, HOP62 and T24 cells, cultured for 72 hours in normal (N) or LR growth conditions. Human liver was used as a positive control for ChREBP detection. Beta-actin was used as a loading control. (**b**) ChREBP translocation to the nucleus was determined by western blot analysis of cytosolic and nuclear fractions of HepG2, PC3M and T24 cells, cultured for 72 hours in normal (N) or LR growth conditions. Total cellular extract of human liver was used as a positive control for ChREBP detection. Alpha-tubulin was and lamin A/C were used as a loading controls.
(TIF)

Figure S5 **Addition of very-low density lipoproteins (VLDL), free fatty acids and cholesterol to lipid reduced (LR) growth conditions reverses the increased expression of SREBP1 and SREBP2 in T24 cell line.** Gene expression levels of SREBP-1a, SREBP-1c and SREBP-2 were analyzed by qPCR analysis in T24 cells cultured for 48 hours in normal (N) or LR growth conditions in the presence or absence of VLDL (**a**), different fatty acid mixtures (**b**) and different concentrations cholesterol (**c**). VLDL was added at a concentration of 607 µg triglycerides/ml serum (corresponding to the concentration triglycerides in normal FBS). Fatty acid (FA) mixtures were as follows, FA Mix 1: 20 µM linoleic (18∶2), 20 µM α-linolenic (18∶3), 5 µM arachidonic (20∶4), 5 µM docosahexaenoic acid (22∶6), FA Mix 2: 10 µM 18∶2, 15 µM 18∶3, 10 µM 20∶4, 15 µM 22∶6 and FA Mix 3: 20 µM 18∶2, 20 µM 18∶3, 5 µM 20∶4, 5 µM 22∶6, 30 µM oleic acid, 30 µM palmitic acid. Different cholesterol (Ch) concentrations are as indicated in the figures (25 µM, 50 µM or 100 µM). Data are normalized to 18S and represented as mean ± S.D. (triplicate per experiment and n = 3). ^*^Significantly different (*p≤0,05; **p≤0,01; ***p≤0,001) from normal medium control. ^#^Significantly different (^#^p≤0,05; ^##^p≤0,01; ^###^p≤0,001) from LR medium control.
(TIF)

Figure S6 **Addition of triglycerides (TG) in combination with recombinant lipoprotein lipase (LPL) to lipid-reduced (LR) growth conditions reverses the increased activation of the lipogenic pathway.** (**a**) T24 cells were cultured for 48 hours in normal (N) or LR growth conditions in the presence or absence of different lipid mixtures. Composition of lipid mixtures were as follows: Mix a: 20 µM linoleic (18∶2) and 20 µM α-linolenic acid (18∶3) and Mix b: 44 µg/ml glyceryltrilinoleate and 44 µg/ml glyceryltrilinolenate. LPL was added at a concentration of 10 µg/ml. During the last 4 hours of culturing ^14^C-acetate was added and the incorporation of radioactivity in cellular lipids was normalized to sample DNA content. Representative experiment is shown, experiment was repeated two times. Significance was determined by one-way ANOVA followed by Tukey's multiple comparison test. *Significantly different (**p≤0,01; ***p≤0,001) from normal medium control. ^#^Significantly different (^###^p≤0,001) from LR control.
(TIF)

Table S1 **Amount of lipids and related components in normal (non-treated) versus lipid-reduced FBS.**
(DOCX)

Table S2 **Ct-values of lipogenic enzymes in different cell lines.** The Ct-values ± STDEV are mentioned of the cell lines cultured in normal and lipid-reduced growth conditions.
(DOCX)
