# Supplementary figures and images for: Cancer Cells Differentially Activate and Thrive on De Novo Lipid Synthesis Pathways in a Low-Lipid Environment
Source: PLoS One. 2014 Sep 12;9(9):e106913. doi: 10.1371/journal.pone.0106913 (PMC4162556; doi:10.1371/journal.pone.0106913)

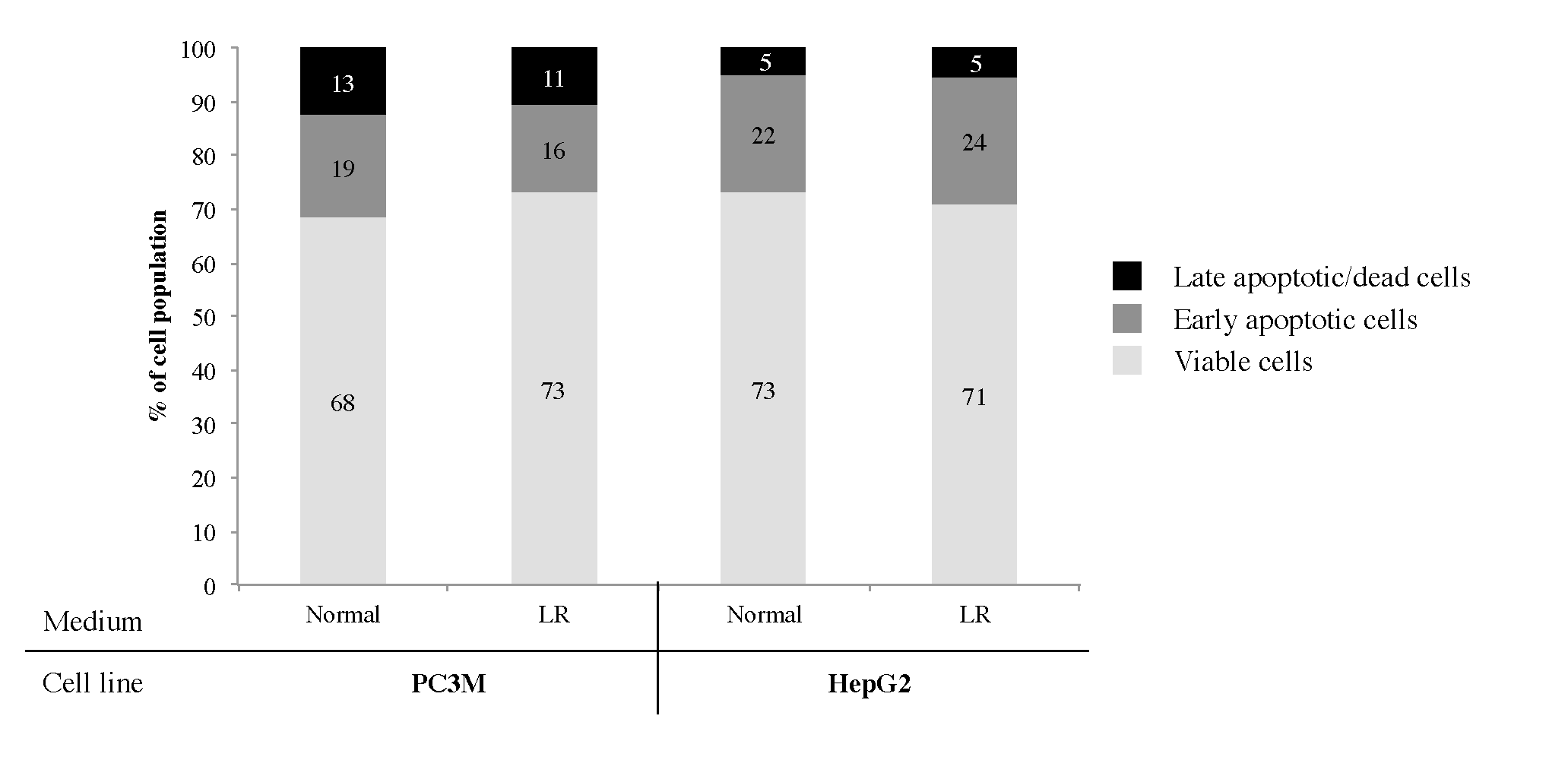

Supplement: Figure S1 — Culturing cells in lipid-reduced (LR) growth conditions does not induce apoptosis. PC3M and HepG2 cells were cultured for 72 hours in normal or LR growth conditions. Cell death was analyzed by flow cytometry as described in materials and methods (n = 3). (TIF) [file pone.0106913.s001.tif]

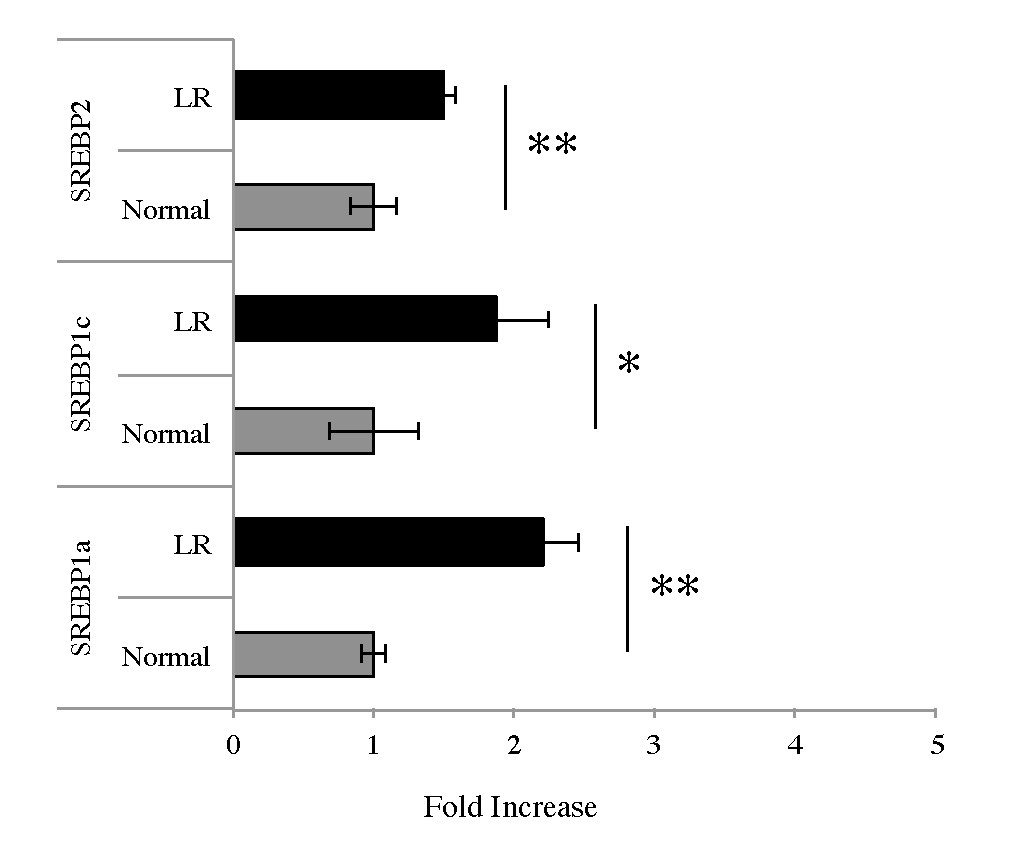

Supplement: Figure S2 — Lipid-reduced (LR) growth conditions increase expression of SREBP1 and SREBP2 in T24 cells. T24 cells were cultured for 48 hours in normal or LR growth conditions. Gene expression levels of SREBP1a, SREBP1c and SREBP2 were analyzed by qPCR analysis. Data normalized to 18S rRNA and represented as mean ± S.D. (triplicate per experiment and n = 3). *Significantly different (*p≤0,05; **p≤0,01; ***p≤0,001). (TIF) [file pone.0106913.s002.tif]

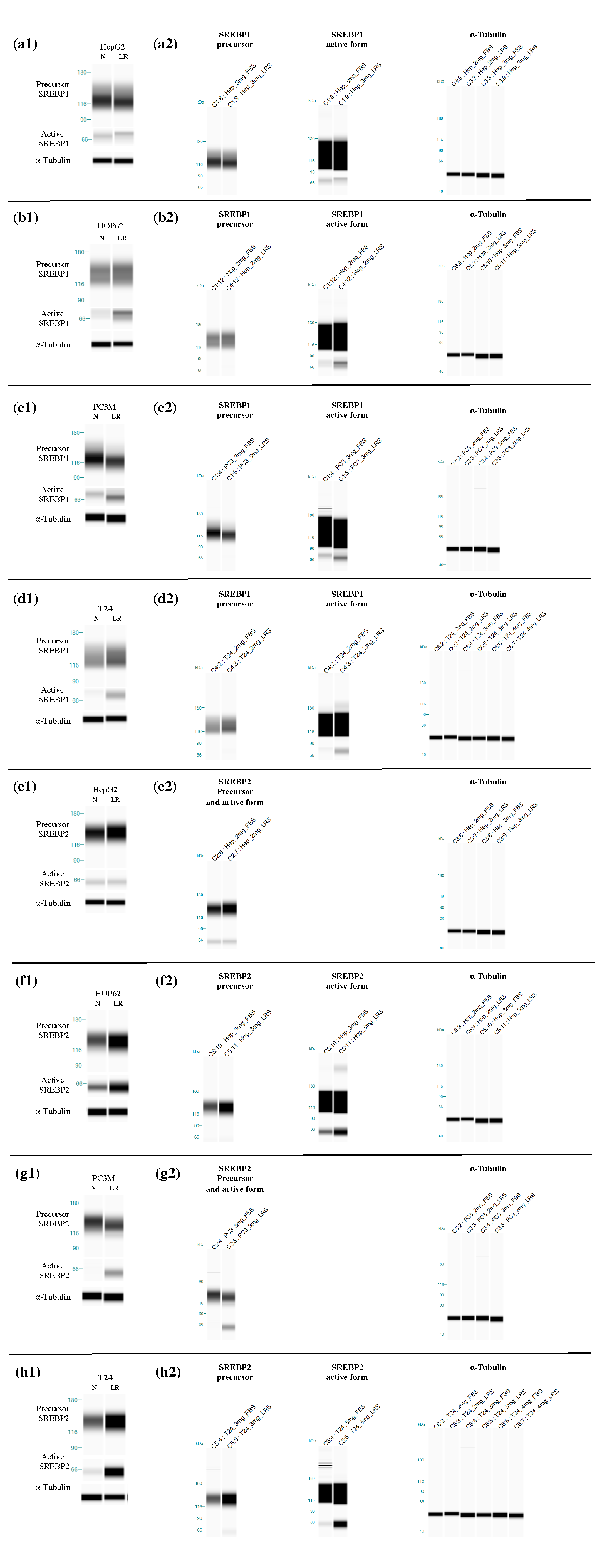

Supplement: Figure S3 — Original data Simple Western analysis of SREBP1 and SREBP2 expression shown in Figure 4a and 4c . Panels shown in Figure 4a and 4c are depicted here in panel 1. They are composed of the original Simple Western bands shown in panel 2. Above the original Simple Western panels is indicated which bands of the blot are used to compose Figure 4a and 4c. The active and precursor SREBP bands shown in Figure 4a and 4c were taken from the same samples, but with a different exposure. This was done in order to have and accurate detection of both the active and the precursor form. Panels (a-d) show composition of panel a of Figure 4 (SREBP1 data), panels (e-h) show composition of panel c of figure 4 (SREBP2 data). (TIF) [file pone.0106913.s003.tif]

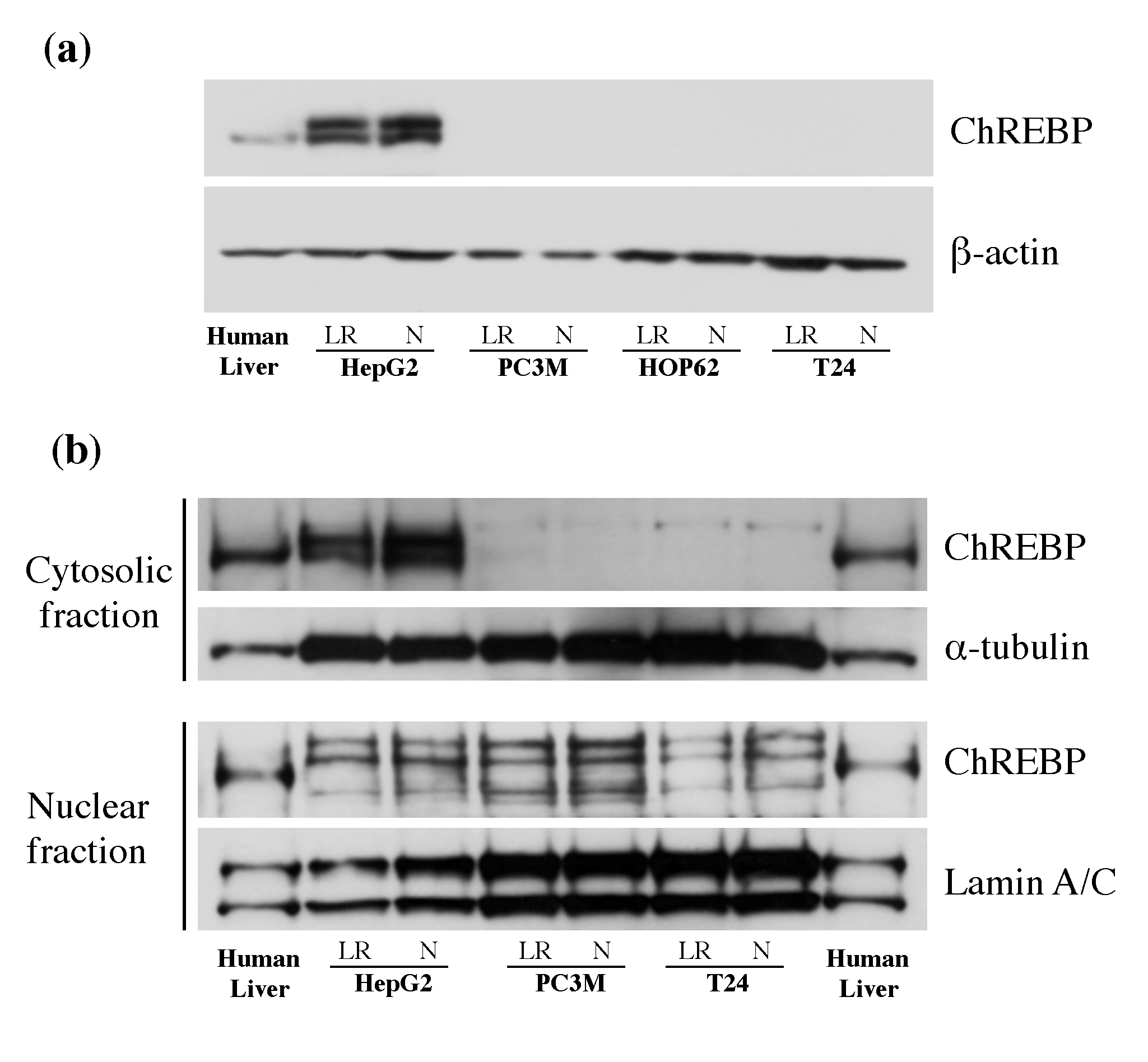

Supplement: Figure S4 — Lipid-reduced (LR) growth conditions do not change the expression and nuclear translocation of ChREBP. (a) ChREBP expression at protein level was analyzed by western blot analysis in HepG2, PC3M, HOP62 and T24 cells, cultured for 72 hours in normal (N) or LR growth conditions. Human liver was used as a positive control for ChREBP detection. Beta-actin was used as a loading control. (b) ChREBP translocation to the nucleus was determined by western blot analysis of cytosolic and nuclear fractions of HepG2, PC3M and T24 cells, cultured for 72 hours in normal (N) or LR growth conditions. Total cellular extract of human liver was used as a positive control for ChREBP detection. Alpha-tubulin was and lamin A/C were used as a loading controls. (TIF) [file pone.0106913.s004.tif]

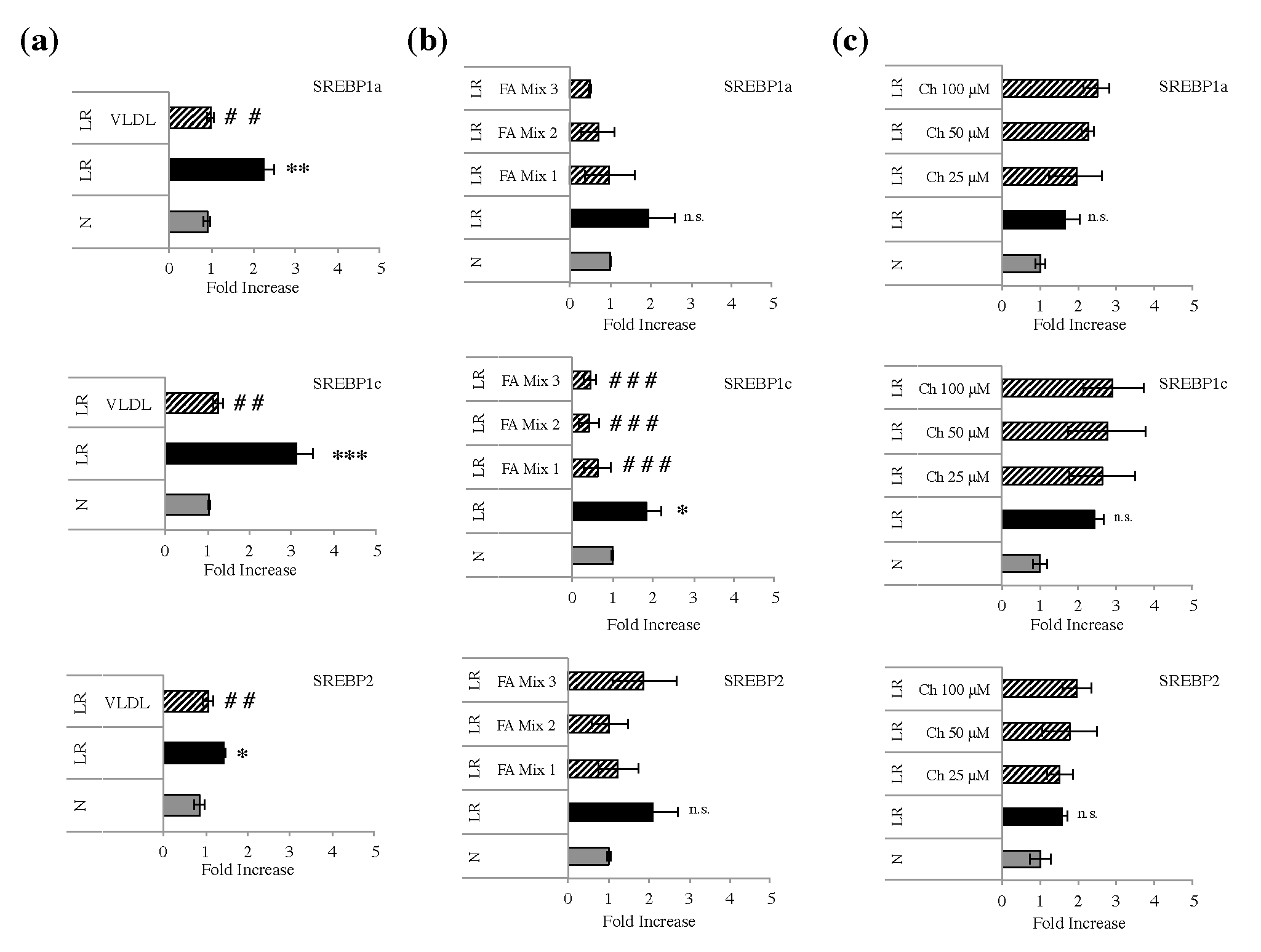

Supplement: Figure S5 — Addition of very-low density lipoproteins (VLDL), free fatty acids and cholesterol to lipid reduced (LR) growth conditions reverses the increased expression of SREBP1 and SREBP2 in T24 cell line. Gene expression levels of SREBP-1a, SREBP-1c and SREBP-2 were analyzed by qPCR analysis in T24 cells cultured for 48 hours in normal (N) or LR growth conditions in the presence or absence of VLDL (a), different fatty acid mixtures (b) and different concentrations cholesterol (c). VLDL was added at a concentration of 607 µg triglycerides/ml serum (corresponding to the concentration triglycerides in normal FBS). Fatty acid (FA) mixtures were as follows, FA Mix 1: 20 µM linoleic (18∶2), 20 µM α-linolenic (18∶3), 5 µM arachidonic (20∶4), 5 µM docosahexaenoic acid (22∶6), FA Mix 2: 10 µM 18∶2, 15 µM 18∶3, 10 µM 20∶4, 15 µM 22∶6 and FA Mix 3: 20 µM 18∶2, 20 µM 18∶3, 5 µM 20∶4, 5 µM 22∶6, 30 µM oleic acid, 30 µM palmitic acid. Different cholesterol (Ch) concentrations are as indicated in the figures (25 µM, 50 µM or 100 µM). Data are normalized to 18S and represented as mean ± S.D. (triplicate per experiment and n = 3). *Significantly different (*p≤0,05; **p≤0,01; ***p≤0,001) from normal medium control. #Significantly different (#p≤0,05; ##p≤0,01; ###p≤0,001) from LR medium control. (TIF) [file pone.0106913.s005.tif]
