# Supplementary material for: Cancer Cells Differentially Activate and Thrive on De Novo Lipid Synthesis Pathways in a Low-Lipid Environment
Source: PLoS One. 2014 Sep 12;9(9):e106913. doi: 10.1371/journal.pone.0106913 (PMC4162556; doi:10.1371/journal.pone.0106913)
Supplement: Table S1 — Amount of lipids and related components in normal (non-treated) versus lipid-reduced FBS. (DOCX) [file pone.0106913.s007.docx]

**Supplementary Table S1:** **Amount of lipids and related components in normal versus lipid-reduced FBS**

|  | **Non-Treated FBS** | **Lipid-Reduced FBS** |
| --- | --- | --- |
| Triglycerols | 766 µg/ml | Not detected |
| Cholesterol | 100 µg/ml | Not detected |
| Ceramides | 37 µg/ml | Not detected |
| Free Fatty Acids | 11 µg/ml | 11 µg/ml |
| Cardiolipins | 191 µg/ml | 200 µg/ml |
| Phosphatidylinositol | Not detected | Not detected |
| Lysophosphatidylserine | 248 µg/ml | 327 µg/ml |
| Phosphatidylcholine | 111 µg/ml | Not detected |
| Sphingomyelin | Not detected | Not detected |
| Phosphatidylserine | Not detected | Not detected |
| Lysophosphatidylcholine | 29 µg/ml | 24 µg/ml |
