# Supplementary material for: Cancer Cells Differentially Activate and Thrive on De Novo Lipid Synthesis Pathways in a Low-Lipid Environment
Source: PLoS One. 2014 Sep 12;9(9):e106913. doi: 10.1371/journal.pone.0106913 (PMC4162556; doi:10.1371/journal.pone.0106913)
Supplement: Table S2 — Ct-values of lipogenic enzymes in different cell lines. The Ct-values ± STDEV are mentioned of the cell lines cultured in normal and lipid-reduced growth conditions. (DOCX) [file pone.0106913.s008.docx]

| Cell Lines | Gene | Cell Culture Medium | |
| --- | --- | --- | --- |
|  |  | Normal | Lipid-Reduced |
| T24 | **FASN** | 19.62 ± 0.33 | 17.58 ± 0.69 |
|  | **ACLY** | 18.51 ± 0.83 | 17.59 ± 0.66 |
|  | **ACSS2** | 23.80 ± 0.34 | 21.88 ± 0.46 |
|  | **HMGCR** | 21.51 ± 0.50 | 19.92 ± 0.44 |
| PC3M | **FASN** | 28.26 ± 0.20 | 27.81 ± 0.88 |
|  | **ACLY** | 27.50 ± 0.12 | 27.32 ± 0.73 |
|  | **ACSS2** | 26.93 ± 0.10 | 25.65 ± 0.75 |
|  | **HMGCR** | 25.14 ± 0.28 | 24.90 ± 0.87 |
| HepG2 | **FASN** | 28.61 ± 0.84 | 28.00 ± 0.64 |
|  | **ACLY** | 27.00 ± 0.69 | 26.54 ± 0.51 |
|  | **ACSS2** | 27.66 ± 0.65 | 26.89 ± 0.45 |
|  | **HMGCR** | 26.98 ±1.02 | 26.57 ± 0.77 |
| HOP62 | **FASN** | 30.60 ± 0.73 | 29.79 ± 0.71 |
|  | **ACLY** | 27.62 ± 0.45 | 27.55 ± 0.51 |
|  | **ACSS2** | 28.55 ± 0.46 | 27.85 ± 0.47 |
|  | **HMGCR** | 28.75 ± 0.60 | 27.86 ± 0.79 |

**Supplemental Table S2: Ct-values of lipogenic enzymes in different cell lines.** The Ct-values ± STDEV are mentioned of the cell lines cultured in normal and lipid-reduced growth conditions.
